# Cheap Medicine for All

**Published:** 1920-11-13

**Authors:** 


### Cheap Medicine For All.

Sir James Cantlie, speaking as a surgeon, has been addressing an interesting little homily to patients in general, which is the same thing as the world in general. The majority of people are perhaps now rid of the old idea that the fixed purpose of a doctor's life, particularly if he is a general practitioner, is first to get patients and then to keep them patients. In other words, it used to be argued, it pays the doctor not to tell a man that his complaint is an imaginary one, or that it is due entirely to his own method of life, and that the cure lies in his own hands. It is rather a stupid tradition which is dying hard, but Sir James Cantlie has managed to give it another much-needed kick by his bold dictum that "walking cures more illnesses than most medicines." His advice may be summed up in the phrase, "Exercise, exercise, exercise." "If you feel seedy, if you are bilious, don't believe that it is hereditary. It is your own fault, and the only way you can remedy it is by exercise." Again, "If your heart palpitates when you go up and down stairs keep on taking exercises until it stops palpitating."

The idea underlying this blunt medical wisdom is a sound one, well worth insisting on at the present day. It is not the doctors who swear by medicines, but the patients. Probably ninety-nine out of every hundred general practitioners in the kingdom could tell from their own experience countless stories of the difficulty of persuading patients that they are not suffering from some mysterious disease or from a specific complaint which can only be cured by taking unpleasant nostrums at stated intervals. The average man seems to regard it as a blow to his self-respect when he is treated in this manner. If he says he is feeling ill, that, in his opinion, should be enough for his doctor, who should take his word for it and make it his business to dispense at once the right drug. It is more, it would seem, than human nature can stand for a man to be told indirectly that he is lazy and must take more exercise. But, as a distinguished doctor told the writer the other day, what is wrong with half the population in these days is that it takes insufficient exercise. The trams, the railways, and the motor-cars are a great convenience to civilisation, "but they are playing ducks and drakes with the health of the people." Few men now will walk a mile if they can possibly ride a mile, and after five years of riding (not on horseback), instead of walking, they are surprised and alarmed because they begin to feel bilious and discover other vague symptoms of incipient

**THE PUBLIC WELL-BEING**—(*continued*).

dyspepsia. It is something to the good that the public generally is growing more interested and better educated in matters affecting the public health; it is, at the same time, curious that individuals, while more self-conscious than they ever were before about the health of their own bodies, persistently follow habits of life which make it almost impossible for them to enjoy that sense of abounding vigour and activity which is the sure sign of perfect health. One of the great problems in our efforts at health reform is not only to provide adequate opportunities of all forms of bodily exercise for the masses of people who are condemned to a sedentary existence during the hours of labour, but to persuade them to take advantage of such opportunities regularly and as a matter of course. The supreme aim in modern medicine is less to cure mankind of the ills that assail it than to prevent avoidable illness, in whatever guise it appears.

